# Antimutagenic Effects of Selenium-Enriched Polysaccharides from *Pyracantha fortuneana* through Suppression of Cytochrome P450 1A Subfamily in the Mouse Liver

**DOI:** 10.3390/molecules21121731

**Published:** 2016-12-16

**Authors:** Fan Peng, Xin Guo, Zhihong Li, Changzheng Li, Changdong Wang, Weiran Lv, Junjie Wang, Fangxiang Xiao, Mohammad Amjad Kamal, Chengfu Yuan

**Affiliations:** 1 Department of Biochemistry, College of Medical Science, China Three Gorges University, Yichang 443002, China; Fanpeng@ctgu.edu.cn (F.P.); guoxin951106@163.com (X.G.); yuancf46@163.com (Z.L.); fangxx@ctgu.edu.cn (F.X.); 2 Nanfang Hospital, Southern Medical University, Guangzhou 510515, China; Changzli@163.com (C.L.); weirl@163.com (W.L.); 3 Molecular Medicine & Cancer Research Center, Chongqing Medical University, Chongqing 400016, China; cdwang@cqmu.edu.cn; 4 Renhe Hospital, China Three Gorges University, Yichang 443002, China; Wangjunjiesx@163.com; 5 King Fahd Medical Research Center, King Abdulaziz University, Jeddah 21589, Saudi Arabia; M.A.kamal@gmail.com; 6 Enzymoics, 7 Peterlee Place, Hebersham, NSW 2770, Australia

**Keywords:** anti-mutagenicity, Se-PFPs, micronucleus formation, CYP1A

## Abstract

Both selenium (Se) and polysaccharides from *Pyracantha fortuneana* (Maxim.) Li (PFPs) (*P. fortuneana*) have been reported to possess antioxidative and immuno-protective activities. Whether or not Se-containing polysaccharides (Se-PFPs) have synergistic effect of Se and polysaccharides on enhancing the antioxidant and immune activities remains to be determined. We previously reported that polysaccharides isolated from Se-enriched *P. fortuneana* (Se-PFPs) possessed hepatoprotective effects. However, it is not clear whether or not they have anti-mutagenic effects. In the present study, we compared and evaluated anti-mutagenic effects of Se-PFPs at three concentrations (1.35, 2.7 and 5.4 g/kg body weight) with those of PFPs, Se alone or Se + PFPs in mice using micronucleus assay in bone marrow and peripheral blood as well as mitomycin C-induced chromosomal aberrations in mouse testicular cells. We also elucidated the underlying mechanism. Our results demonstrated that Se-PFPs inhibited cyclophosphamide (CP)-induced micronucleus formation in both bone marrow and peripheral blood, enhanced the activities of superoxide dismutase (SOD) and glutathione peroxidase (GPx) in mouse liver, and reduced the activity and expression of cytochrome P450 1A (CYP4501A) in mouse liver in a dose-dependent manner. In addition, we found that the anti-mutagenic potential of Se-PFPs was higher than those of PFPs, Se alone or Se + PFPs at the same level. These results suggest that the anti-mutagenic potential of Se-PFPs may be mediated through the inhibition of the activity and expression of CYP4501A. This study indicates that application of Se-PFPs may provide an alternative strategy for cancer therapy by targeting CYP1A family.

## 1. Introduction

The genetic changes in cancer cells or the mutations of cancer-related genes, including point mutation of oncogenes and cancer suppressor genes, insertion or deletion of small DNA fragment, DNA rearrangement, copy number variation, acquisition of exogenous DNA segment, etc. have been causally associated with oncogenesis [1,2,3]. Up to July 2010, the Catalogue of Somatic Mutations in Cancer (COSMIC) had described a total of 136,326 coding mutations among 541,928 tumor samples where 26% (4803/18,490) of the documented genes had at least one mutation [4]. In addition, the correlations between the mutation of 5′-untranslated region (5′-UTR), 3′-UTR region or promoter in mRNA and tumorigenesis have been documented [5,6,7]. Genomic instability is one of the most prominent characteristics of tumor cells, and is probably the comprehensive consequence of DNA damage, DNA repair-deficiency and regulation failure of cell cycle checkpoints [8,9]. There exist certain relationships between DNA damage/mutation and oxidative stress. Increasing lines of evidence have suggested that the development and progression of several types of diseases, including cancer, neurodegenerative diseases, diabetes, autoimmune disorders, etc. are significantly impacted by oxidative stress. The pathogenic impacts of oxidative stress are mainly mediated through chromatin alterations and cellular changes, which are the common mechanisms for those human pathologies [10,11,12,13]. Therefore, oxidative stress is a promising target for the prevention and treatment of these diseases.

Cyclophosphamide (CP) is an alkylating agent with chemotherapeutic activity. Its alkyl group is attached to DNA and interferes with DNA replication by forming intra- and inter-strand DNA crosslinks. On the other hand, CP is also carcinogenic and may increase the risk of developing lymphomas, leukemia, skin cancer, transitional cell carcinoma of the bladder and other malignancies [14]. Both in vivo and in vitro studies have found that CP can induce genetic instabilities during the later developmental stage by disturbing epigenetic programming, induce embryonic ocular malformations, cause birth defects and induce embryotoxic and teratogenic changes [15,16,17,18,19]. Orally administered CP is rapidly absorbed and then converted to active metabolites by cytochrome P450s (CYP450s) in the liver [20]. Thus, it is reasonable to hypothesize that the cytotoxic effects of CP could be suppressed through inhibition of CYP450 pathway.

*Pyracantha fortuneana* (Maxim.) Li (*P. fortuneana*) is a species of firethorn and mainly distributed in Europe, China and Vietnam. Its fruit can be applied as a traditional Chinese medicine to treat dyspepsia and dysentery [21]. *P. fortuneana* contains a variety of functional components, including flavonoids, polysaccharides, pigments, polyphenols and phospholipids, with several medical and pharmaceutical functions, including tyrosinase inhibitory activity, antioxidant and anti-fatigue activities, anti-bacterial activity and cancer prevention [22]. Our previous studies have shown that polysaccharides from *P. fortuneana* (PFPs) possess anti-oxidative and immuno-protective activities [23,24,25]. Several studies by other investigators have also demonstrated that selenium (Se), an important antioxidant, has chemoprotective and anti-carcinogenic effects [26,27,28,29]. Se can prevent the transformation of cells with genomic mutations into neoplastic cells by promoting the antioxidant capacity and immunological response as well as inhibiting the activities of the key enzymes involved in carcinogenesis [30]. Se also has anti-proliferative and cytotoxic effect on myelodysplastic cells by inducing apoptosis mainly due to induction of reactive oxygen species (ROS) [31]. Polysaccharides from plants have been evaluated for their anti-tumor activities, in which the direct activities included induction of apoptosis of tumor cells, arrest of its cell cycle and inhibition of its invasion, adhesion and metastasis while the indirect activities included enhancement of the immune protection [32,33,34]. Recent studies have indicated that Se-containing polysaccharides provide the effects of reducing oxidative stress and antitumor immunomodulation [35,36]. Our previous research reported that polysaccharides derived from Se-enriched *P. fortuneana* had hepatoprotective effects [23,24,25]. We also found that it could potently inhibit the growth of breast cancer MDA-MB-231 cells [37]. Therefore, it is reasonable to hypothesize that Se-containing polysaccharides may have synergistic effect of Se and polysaccharides on enhancing the antioxidant and immune activities. This study aimed to test this hypothesis by evaluating the in vivo anti-mutagenic effects of Se-containing polysaccharides isolated from Se-enriched *P. fortuneana* and comparing their effects to those of PFPs, Se and Se + PFPs in mice.

## 2. Results

### 2.1. Anti-Mutagenic Effects of Se-PFPs in Mice

We reported previously that PFPs possessed antioxidative and immunoprotective activities and that Se-PFPs possessed hepatoprotective effect [23,24]. Therefore, the influences of Se-PFPs, PFPs, Se or PFPs + Se on mice were analyzed to evaluate the anti-mutagenic effect of Se-PFPs in the present study. The mean initial body weight, mean final body weight, mean body weight gain and mean liver index of mice treated with Se-PFPs, PFPs, Se or PFPs + Se plus or minus CP were presented in Table 1. During the period of treatment, mice in all the administered groups showed the increase in mean body weight and a slight reduction in mean body weight gain in CP-treated group as compared to negative control group but no statistically significant differences in mean body weight gain among these groups were found (*p* > 0.05). Though a slight increase in liver index in CP-group and other treatment groups compared to that of negative control group, but no statistically significant differences in liver index of mice among these groups were also found (*p* > 0.05), suggesting that the toxic effects of Se-PFPs treatment to mice is not detectable during this period of treatment.

Bone marrow micronucleus (MN) assay was performed to analyze the in vivo anti-mutagenic effect of Se-PFPs in mice. As shown in Table 2, compared with negative control, Se-PFPs, PFPs, Se alone or PFPs + Se groups showed no significant increase in MN formation (*p* > 0.05) whereas CP alone significantly induced MN formation in the bone marrow (*p* < 0.05). CP-induced MN formation was reduced by 57.8%, 73.9% and 86.3% in mice after treatments with Se-PFPs at 1.35, 2.7 and 5.4 (g/kg·BW), respectively, the percentages of reduction were clearly and significantly increased with increasing Se content (*p* < 0.05). The administration of PFPs, Se, or PFPs + Se also significantly inhibited CP-induced MN formation in bone marrow by 41.7%, 44.2% and 62.6%, respectively (*p* < 0.05). Furthermore, Se-PFPs caused significantly higher inhibition on CP-induced MN formation in bone marrow than did PFPs, Se or PFPs + Se at the same level (*p* < 0.05), suggesting that the anti-mutagenic effect of Se-PFPs is higher than those of PFPs, Se, or Se + PFPs at the same level.

The results of MN assay in peripheral blood of mice were presented in Table 3, which were quite similar to those in bone marrow. No significant differences in micronucleated reticulocytes (MNRETs) were found among Se-PFPs, PFPs, Se alone, or PFPs + Se groups (*p* > 0.05) while MNRETs were significantly increased in CP alone (*p* < 0.05). CP-induced MN formation in peripheral blood was significantly inhibited by co-administration with Se-PFPs at 1.35, 2.7 and 5.4 (g/kg·BW) by 56.8%, 73.2%, and 87.9% inhibition, respectively with the increasing Se content (*p* < 0.05). The co-administration of PFPs, Se, and PFPs + Se also significantly inhibited CP-induced MN formation in peripheral blood, with 40.7%, 43.5% and 61.0% reduction, respectively (*p* < 0.05). Moreover, Se-PFPs caused a significantly higher inhibition on CP-induced MN formation in peripheral blood than did of PFPs, Se and PFPs + Se at the same level (*p* < 0.05). To further confirm these results, we also examined the effects of Se-PFPs, PFPs, Se and PFPs + Se on mitomycin C (MMC)-induced chromosomal aberrations in mouse testicular cells. The results are presented in Supplementary Materials Table S1, which also show that MMC-induced chromosomal aberrations in mouse testicular cells was significantly inhibited by treatment of mice with Se-PFPs at 1.35, 2.7 and 5.4 g/kg·BW, with 54.2%, 72.1% and 92.5% of inhibition, respectively. Similarly, MMC-induced chromosomal aberrations in mouse testicular cells were also significantly inhibited by PFPs, Se, and PFPs + Se (*p* < 0.05), with 41.7%, 44.2% and 62.6% of inhibition, respectively. Again, Se-PFPs caused significantly higher inhibition than did PFPs, Se, or PFPs + Se at the same level. Together, these results clearly indicate that Se-PFPs have significantly higher anti-mutagenicity in mice.

### 2.2. Antioxidative Activity of Se-PFPs in Liver of Mice 

To determine whether the anti-mutagenic potential of Se-PFPs is associated with anti-oxidant and anti-carcinogenicity, we measured the activities of superoxide dismutase (SOD) and glutathione peroxidase (GPx) in the liver of mice treated with Se-PFPs, PFPs, Se, or + PFPs + Se. The results in Table 4 revealed that activities of both SOD and GPx were significantly decreased after CP treatment by approximately 2.4 folds (*p* < 0.05). Compared with CP treatment, co-administration of CP with Se-PFPs at 1.35, 2.7 and 5.4 g/kg·BW significantly increased the activity of SOD (131.4 ± 16.0, 168.1 ± 5.0 and 185.6 ± 9.2 U/mg, respectively) and the activity of GPx (157.7 ± 19.2, 198.9 ± 15.2 and 22.8 ± 10.9 U/mg, respectively) (*p* < 0.05). Activities of SOD and GPx were significantly increased with the increasing Se-PFPs content in a dose-dependent manner (*p* < 0.05). Co-administration of mice with CP plus PFPs, Se, or PFPs + Se also significantly increased the activities of SOD (132.4 ± 9.3, 127.7 ± 12.8 and 152.6 ± 12.9, respectively) and the activities of GPx (158.9 ± 12.4, 149.2 ± 10.5 and 183.1 ± 15.5, respectively) as compared to that of SOD (83.2 ± 7.9) and that of GPx (99.7 ± 12.1) of CP-treatment alone. It was noted that Se-PFPs also induced significantly higher activities of SOD and GPx than did PFPs, Se and PFPs + Se at the same level (*p* < 0.05).

### 2.3. Influence of Se-PFPs on Heaptic Cytochrome P450 1A (CYP1A)

Many exogenous carcinogens can be metabolized to their ultimate carcinogenic forms by cytochrome P450s (CYPs), especially the members of CYP1A family [38]. CYP1A is involved in the activation of many carcinogens, generation of electrophilic intermediate or the end products and interaction with nucleophilic group of macro-molecules, resulting in the changes in cellular structure, inactivation or abnormality of enzymes, induction of DNA damage or inhibition of gene expression, ultimately leading to cellular damage, apoptosis or tumor formation. CYP1A1 is widely distributed in the extra-hepatic tissues, such as lung, kidney, skin, lymphocytes and brain, accounting for 2.5% of the total xenobiotic metabolism. CYP1A2 is specifically expressed in liver and shares 68% homology with CYP1A1, accounting for 8.2% of xenobiotic metabolism [39]. Hence, in the present study, the activities of CYP1A1 and CYP1A2, and the expression of CYP1A in the liver of mice treated with Se-PFPs, PFPs, Se and PFPs + Se in the presence and absence of CP were detected in order to understand mechanisms underlying anti-mutagenicity of Se-PFPs.

The activities of 7-ethoxy resorufin deethylase (EROD) and methoxyresorufin-*O*-deethylase (MROD) can represent the activity of CYP1A1 and the activity of CYP1A2, respectively. Table 5 reveals that the activity of EROD displayed no significant difference between control (38.17 ± 1.95 pmol/min/mg protein) and CP group (38.10 ± 3.18 pmol/min/mg protein) (*p* > 0.05) and the activity of MROD also displayed no significant difference between negative control (61.27 ± 9.29 pmol/min/mg protein) and CP group (61.1 ± 7.93 pmol/min/mg protein) (*p* > 0.05), suggesting that CP itself has no significant effect on either EROD or MROD activity. Compared with CP treatment, the administration of Se-PFPs, PFPs, or PFPs + Se significantly reduced the activity of EROD (*p* < 0.05). Specifically, the administration of Se-PFPs significantly reduced the activity of EROD in a dose-dependent manner. Furthermore, the inhibitory effect of Se-PFPs + CP on EROD activity (20.97 ± 2.06) was more potent than those of PFPs + CP (28.57 ± 1.51), and PFPs + Se + CP (28.73 ± 2.41) at the same level. Similarly, compared with CP treatment, the administration of Se-PFPs, PFPs, or PFPs + Se significantly reduced MROD activity (*p* < 0.05). Administration of Se-PFPs significantly reduced MROD activity in a dose-dependent manner, i.e., EROD activity was reduced from 38.10 ± 3.18 (CP-group) to 28.93 ± 2.36, 20.97 ± 2.06, and 13.13 ± 3.56 by co-administration of Se-PFPs at 1.35, 2.7 and 5.4 g/kg·BW, respectively. MROD activity was reduced from 61.1 ± 7.93 (CP-group) to 44.00 ± 5.03, 26.93 ± 2.68 and 10.00 ± 1.77 by co-administration with Se-PFPs at 1.35, 2.7 and 5.4 g/kg·BW, respectively. Furthermore, the inhibitory effect of Se-PFPs at on EROD activity (20.97 ± 2.06) was more potent than those of PFPs + CP (28.57 ± 1.51), and PFPs + Se (28.73 ± 2.41) at the same level. Similarly, the inhibitory effect of Se-PFPs on MROD activity (26.93 ± 2.68) was more potent than those of PFPs (43.88 ± 3.45). However, Se alone had no significant effect on the activities of both EROD and MROD (*p* > 0.05).

**Figure 1:**
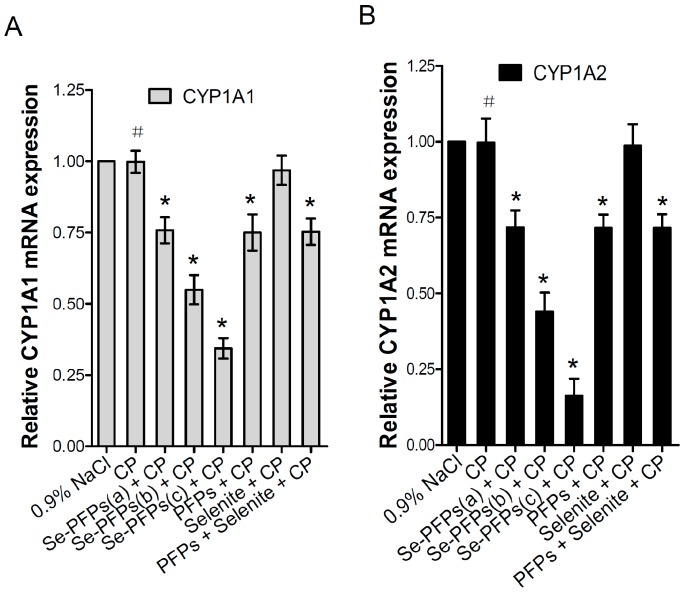
Effects of administration of Se-PFPs, PFPs, selenite, and PFPs + selenite on relative mRNA expression levels of: CYP1A1 (**A**); and CYP1A2 (**B**) relative to GAPDH in liver microsomes of mice examined by RT-PCR. All the values are shown as means ± SD (*n* = 10). ^#^ *p* > 0.05, compared with 0.9% NaCl; * *p* < 0.05, compared with CP. PFPs, polysaccharide from *P. fortuneana*, Se-PFPs, polysaccharides from Se-enriched *P. fortuneana*, and PFPs + selenite, the combination of PFPs and selenite.

The mRNA and protein levels of CYP1A1 and CYP1A2 in the liver microsome of mice were further evaluated using RT-PCR and Western blot, respectively. As shown in Figure 1A, no significant difference in mRNA level of hepatic CYP1A1 was found between CP and the negative control group (*p* > 0.05). Se-PFPs significantly reduced the mRNA level of hepatic CYP1A1 in a dose-dependent manner (*p* < 0.05). PFPs and PFPs + Se also reduced mRNA level of hepatic CYP1A1 (*p* < 0.05) while Se alone had no significant effects on them (*p* > 0.05). Se-PFPs treatment decreased more mRNA level of hepatic CYP1A1 than PFPs and Se + PFPs did at the same level (*p* < 0.05). As shown in Figure 1B, no significant difference at mRNA level of hepatic CYP1A2 in CP treatment was found (*p* > 0.05) as compared with that of the negative control. Se-PFPs significantly reduced mRNA level of hepatic CYP1A2 in a dose-dependent manner (*p* < 0.05). PFPs and PFPs + Se also significantly reduce mRNA level of hepatic CYP1A2 (*p* < 0.05) while Se alone had no significant influence on mRNA level of CPY2 (*p* > 0.05). Se-PFPs decreased more mRNA level of hepatic CYP1A2 than that did PFPs and PFPs + Se at the same level (*p* < 0.05).

**Figure 2:**
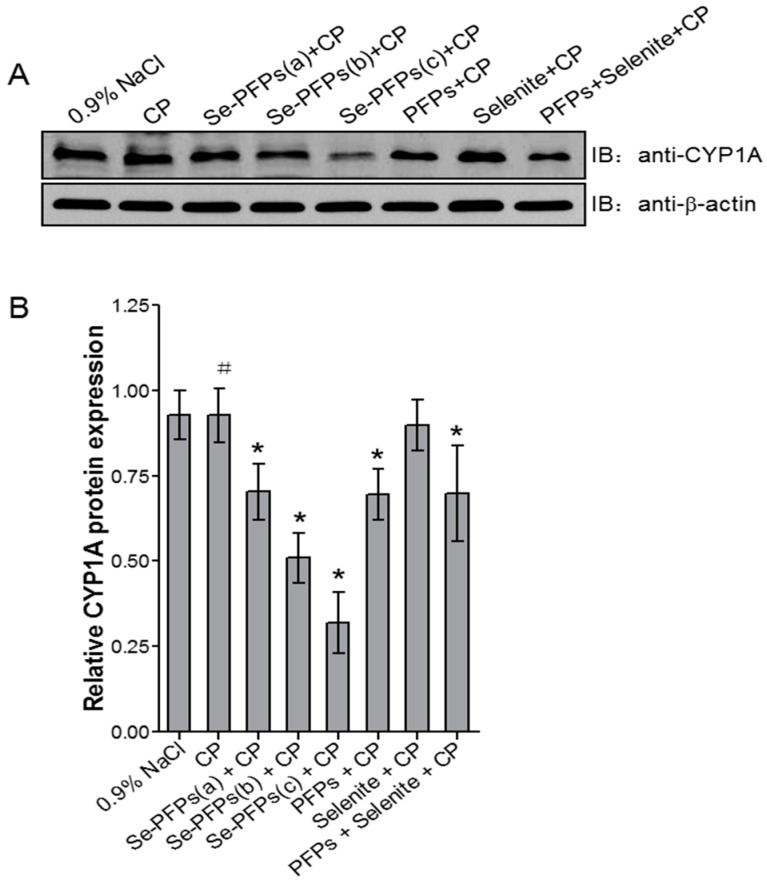
Effects of administration of Se-PFPs, PFPs, selenite, and PFPs + selenite on relative protein levels of CYP1A in mouse liver microsome by Western blotting: (**A**) representative bands showing the changes in CYP1A protein levels after different treatments; and (**B**) the protein levels of CYP1A in relative to that of β-actin of corresponding treatments. All the values are shown as means ± SD (*n* = 10). ^#^ *p* > 0.05, compared with 0.9% NaCl; * *p* < 0.05, compared with CP. PFPs, polysaccharide from *P. fortuneana*, Se-PFPs, polysaccharide from Se-enriched *P. fortuneana*, and PFPs + selenite, the combination of PFPs and selenite.

The effects of various treatments on protein level of hepatic CYP1A1 were examined and the results were shown in Figure 2. Figure 2A showed the representative Western blot analysis of protein level of hepatic CYP1A and Figure 2B presented the quantitative data based on Western blot analysis. No significant difference in protein level of hepatic CYP1A was found between negative control and CP treatment (*p* > 0.05). Se-PFPs treatment significantly reduced protein level of hepatic CYP1A in a dose-dependent manner (*p* < 0.05) (Figure 2A,B). PFPs and PFPs + Se also significantly reduced protein level of hepatic CYP1A (*p* < 0.05) whereas Se alone had no significant effect on it (*p* > 0.05). Se-PFPs decreased more protein level of hepatic CYP1A than did PFPs and PFPs + Se at the same level (*p* < 0.05).

The more intriguing point is that Se-PFPs treatment can clearly reduce both the activity and expression of hepatic CYP1A in a dose-dependent manner in the present study. PFPs or PFPs + Se could also clearly reduce both the activity and expression of hepatic CYP1A while Se alone had no effect on them. Meanwhile, the reduction of Se-PFPs administration is stronger than that of PFPs, Se or PFPs + Se at the same level. The results suggest that the inhibition of hepatic CYP1A activity and expression is associated to anti-mutagenicity of Se-PFPs, which may provide an alternative strategy for cancer therapy by targeting the relationship between Se-PFPs and CYP1A.

## 3. Discussions

We found previously that polysaccharides isolated from Se-enriched *P. fortuneana* (Se-PFPs) possessed hepatoprotective effects [23,24]. Administration of PFPs significantly increased thymus and spleen indexes, promoted proliferation of splenocyte and natural killer (NK) cell activity, and elevated numbers of CD4 T cells as well as CD4(+)/CD8(+) ratios. PFPs also increased interleukin-2 (IL-2) levels, and decreased IL-6 level and tumor necrosis factor-alpha (TNF-α) in splenocytes [23]. In a subsequent study, we also observed that Se-PFPs conferred preventive effects against carbon tetrachloride (CCL4)-induced liver injury through enhancing the activities of SOD and GPx and glutathione (GSH) level in a mouse model [24]. These results indicate that Se-PFPs possessed hepatoprotective effects [23,24]. In the present study, we further evaluated the in vivo anti-mutagenic effects of Se-PFPs in mice and made several interesting findings, including administration of mice with Se-PFPs: (A) significantly inhibited CP-induced mutagenicity; (B) enhanced the antioxidant system in mouse liver; and (C) reduced the activities of CYP1A1 and CYP1A2 in mouse liver, and mRNA and protein levels of CYP1A in mouse liver microsomes in a dose-dependent manner. Moreover, we also found that Se-PFPs caused significantly higher inhibition on CP-induced MN formation in bone marrow, induced significantly higher activities of SOD and GPx, and caused significantly stronger inhibition on the CP-induced mutagenicity; more significantly, it down regulated the expression of mRNA and protein level of hepatic CYP1A1 and CYP1A2 as compared to those induced by PFPs and PFPs + Se at the same level, respectively.

The in vivo anti-mutagenic effects of Se-PFPs in mice are indicated by their inhibition on CP-induced micronucleated polychromatic erythrocytes (MNPCEs) and MNRETs in mice. CP can generate free radicals and attachment of its alkyl group to DNA, interferes with DNA replication by forming intra- and inter-strand DNA crosslinks alkylate DNA, causing damage to chromosomes and inducing mutagenicity [40]. Thus, CP is also carcinogenic. A micronucleus test, which is widely used in screening for potential genotoxic compounds, has been regarded as one of the most reliable assays for genotoxic carcinogens. Mouse bone marrow and/or mouse peripheral blood are used for the in vivo test due to the reason that when a bone marrow erythroblast develops into a polychromatic erythrocyte, the main nucleus is extruded; any micronucleus that has been formed may remain behind in the otherwise anucleated cytoplasm [41]. An increase in the frequency of micronucleated polychromatic erythrocytes in treated animals is an indication of induced chromosome damage [41]. In this study, we observed that CP-induced MNPCEs and MNRETs in mice were significantly inhibited by Se-PFPs in a dose-dependent manner, clearly indicating that the anti-mutagenic effects of Se-PFPs in mice.

The protective role of Se on genetic damage and on cancer has been recognized [40]. Se is an essential dietary component for humans and other animals. It acts as a protective agent against cancer. Its protective effects are mediated via specific inhibition of tumor cell growth by its metabolites, antioxidant protection by selenoenzymes, regulation of cell cycle and apoptosis and effect on DNA repair [42,43,44]. Previous study had indicated that super-nutritional Se level (>5 μmol/L) induced apoptosis of cancer cells mainly due to ROS induction [45]. It is found that selenite can induce apoptosis in NB4 cells and cell cycle arrest at G0/G1 phase through ROS/JNK/ATF2 pathway [46]. The anti-tumor activities of polysaccharides extracted from plants such as *Sophora flavescens* Ait [32], the roots of *Polygala tenuifolia* [33] and *Boschniakia rossica* [34] have been evaluated. Their direct activities include induction of apoptosis of tumor cell, arrest of its cell cycle and inhibition of invasion, adhesion and metastasis, and their indirect activities include enhancement of the immune protection [32,33,34]. In our previous researches [23,24,25], we found that PFPs possessed antioxidative and immunoprotective activities. In the present study, we found that Se-PFPs significantly inhibited CP-induced MNPCEs and MNRETs in mice in a dose-dependent manner and also significantly inhibited MMC-induced chromosomal aberrations in mouse testicular cells. Together, these results clearly indicate the potential of Se-PFPs as an anti-mutagenic agent.

To elucidate the mechanisms underlying the anti-mutagenetic effects of Se-PFPs, we examined the effects of Se-PFPs on the activities of two important antioxidant system-related enzymes, SOD and GPx. We found that administration of mice with Se-PFPs significantly improved the activities of both SOD and GPx in mouse liver in a dose-dependent manner. These results are consistent with our previous results [23,24,47]. In our previous study [23], treatment of mice with PFPs resulted in substantial increases in the activities of SOD and GPx, increase the mRNA and protein expression levels of nuclear factor erythroid 2-related factor (Nrf2), a transcriptional factor that regulates the expression of antioxidant proteins, accompanied by a dramatic decrease in malondialdehyde (MDA) levels in mouse splenocytes. In our another study [24], we observed that treatment of mice with Se-PFPs protected CCL4-induced liver injury via increasing mRNA and protein expression levels of GPx and catalase (CAT) in liver and decreased the levels of thiobarbituric acid reactive substances and H_2_O_2_. Moreover, in a more recent study, we observed that a combination of selenium-enriched green tea polysaccharides with Huo-ji polysaccharides synergistically enhanced antioxidant and immune activity in mice via reducing the levels of inflammatory cytokines, such as TNF-á and IL-6, remarkably enhancing the activities of GPx and SOD, significantly increasing mRNA and protein expression levels of Nrf2 and significantly reducing MDA levels in mice in splenocytes [47]. SOD, an enzyme that alternately catalyzes the dismutation of the superoxide (O^2−^) radical, the most dangerous ROS with a potential leading to lipid peroxidation and oxidation of DNA and proteins, into either molecular oxygen (O_2_) or H_2_O_2_, is a key antioxidant defense in almost all living cells against ROS-induced oxidative damage [48,49]. GPx, an enzyme catalyzing the reduction of a variety of hydroperoxides (e.g., ROOH and H_2_O_2_) using glutathione, is another important antioxidant involved in protecting mammalian cells against oxidative damage [48], indicating that Se-PFPs-mediated induction of the activities of SOD and GPx in liver by Se-PFPs can play the key roles in the enhancement of anti-mutagenicity of Se-PFPs.

An intriguing finding made in this study is that treatment of mice with of Se-PFPs significantly reduced the activities of CYP1A1 and CYP1A2 in mouse liver microsomes, and mRNA and protein levels of CYP1A in mouse liver in a dose-dependent manner. The members of CYP450 family play critical roles in the biotransformation of carcinogens, steroid hormones, drugs and environmental substances [50,51]. CYP1A1 and CYP1A2 are capable of catalyzing the oxygenation of heterocyclic aromatic amines/amides (HAAs) and polycyclic aromatic hydrocarbons (PAHs), the dealkylation of phenacetin, demethylation of aminoazo dyes, and other therapeutic agents [52,53]. Both CYP1A1 and CYP1A2 catalyze the oxygenation of the chemicals, which represents an initial step in the conversion of the substrates to more polar metabolites, leading to the increased excretion and maintaining the chemical homeostasis in the body. However, when the carcinogenic PAH and HAA (procarcinogen) are oxygenized by CYP1A1 and CYP1A2 to produce arene oxide, diolepoxide, and other electrophilic reactive species (ultimate carcinogen) that form DNA and protein adducts, they can result in tumor formation and toxicity. A critical step in cancer formation in human populations exposed to PAHs and HAAs is the metabolic activation of PAHs and HAAs by members of CYP450 1A family [53]. The expression at both mRNA and protein levels of CYP1A1 and CYP1A2 is highly inducible by a variety of chemicals [54]. It has been known that CYP1A1 is normally expressed at low levels in extrahepatic tissues in humans but is highly inducible in the liver and extrahepatic tissues, while CYP1A2 is constitutively expressed in the liver and is inducible. PAH-induced expression of CYP1A is mediated through the aryl hydrocarbon receptor (AhR), a ligand-activated transcription factor [53]. Because the metabolic activation of PAHs and HAAs by CYP1A1 and CYP1A2 will ultimately leads to carcinogens, it is likely that induction of these enzymes is detrimental in humans exposed to high levels of PAHs and HAAs. It has been known that orally administrated CP is rapidly absorbed and then converted by mixed-function CYP450 system to active metabolites in the liver [20]. The significant reduction of activities of CYP1A1 and CYP1A2 in mouse liver microsomes and the reduction of mRNA and protein levels of CYP1A1 can substantially reduce the CYP450-mediated conversion of CP to active metabolites in liver and ultimately reduce the CP-induced genomic mutagenicity. In addition to CYP1A1 and CYP1A2, other CYP enzymes may be also involved in inhibition of carcinogen-induced genomic mutagenicity. For instance, CYP2B6 was reported to be involved in CP catalyzation, while the activities of CYP1A1, 1A2, 2D6 and 2El were undetectable [55]. CYP2C9 was found to be the major CYP isoform responsible for CP hydroxylation at low concentration (0.1 mmol/L) [56]. In another study, we also found that the anti-mutagenic potential of Se-PFPs may be involved in the inhibition of expression of CYP2B6 and CYP2C9 in mouse liver in a dose-dependent manner (unpublished data). Thus, it appears that Se-PFPs are capable of down-regulating a number of CYP450 enzymes and thus, inhibiting CYP450-catalyzed conversion of pro-carcinogens and carcinogens to active carcinogenic metabolites, and ultimately reducing the genomic mutagenicity and carcinogenesis.

We observed that Se-PFPs, PFPs and PFPs + Se were capable of inhibiting the activity and expression of CYP1A1 and CYP1A2 but Se itself did not cause significant effects, indicating that the PFPs moiety of Se-PFPs is responsible for the inhibitory effects of Se-PFPs on CYP1A1 and CYP1A2. This is consistent with the previous observation that polysaccharides isolated from the edible and medicinal mushrooms, *Lentinus edodes* and *Agaricus blazei*, suppressed the expression of cytochrome P450s in mice [38]. While the molecular mechanisms underlying the regulation of CYP450 enzymes by Se-PFPs is not clear at present, it is known that aryl hydrocarbon receptor (AhR), the key transcriptional factors, is involved in transcriptional regulation of CYP450 genes, and the interactions between AhR and inflammatory signaling evidently can play a significant role in immune dysfunctions, metabolism of xenobiotics or carcinogenesis [39]. It is possible that the polysaccharides may serve as the competitive inhibitor competing with CP- and/or other ligands for the binding to AhR. In our previous study, we found that administration of mice with a combination of selenium-enriched green tea polysaccharides with Huo-ji polysaccharides synergistically reduced the levels of inflammatory cytokines, such as TNF-α and IL-6 [47]. It has been known that inflammatory mediators play important roles in regulation of CYPs [57]. The effects of Se-PFPs on expression of CYP1A1 and CYP1A2 may be also mediated, at least in part, by inflammatory cytokines. However, the precise mechanisms underlying Se-PFPs-mediated down regulation of CYP450 enzymes need to be further elucidated.

Another intriguing finding made in this study is that the effects of Se-PFPs on inhibition of CP-induced mutagenicity (EROD or MROD activities or MMC-induced chromosomal aberrations), induction of activities of SOD and GPx, suppression of activities of CYP1A1 and CYP1A2, and suppression of CYP1A1 expression were all significantly higher than those of PFPs, Se or PFPs + Se at the same concentration. These results indicate the synergetic effects of Se-PFPs on these cellular responses over those of the individual PFPs or along and the combination of PFPs + Se. Consistent with this finding, in in vitro studies have indicated that the dose and form of selenium compounds are the critical factors determining the cellular responses. For instance, inorganic Se (at doses > 10 μmol/L) and organic Se-containing compounds (at doses ≥ 10 μmol/L) can elicit distinctly different cellular responses [40]. The dose and the form of selenium are the critical factors in cancer prevention. For instance, three forms of selenium, namely sodium selenite, l-selenomethionine (SeMet), and methyl l-selenocysteine (MSC) are known to be the most important in cancer prevention. They differ in the way by which they are handled inside the body, and thus, differ in their impact on the risk for cancer [58]. It is likely that different chemical forms of Se are intracellularly metabolized via complicated pathways, which target distinct molecules and exhibit varying degrees of anti-carcinogenicity in different cancer types. It was reported that the organic selenium compound SeMet is better absorbed than inorganic sodium selenite [59]. Suzuki et al. [60] reported that different organoselenium compounds, i.e., MSC, SeMet, and Se caused differential apoptotic response of human cancer cells, in that Se-induced apoptosis in carcinoma cells is basically a caspase-dependent process involving the activation of both the intrinsic apoptotic pathway and ER stress pathway, while SeMet-induced apoptosis appears to be mediated via p53 activation. The better effects of Se-PFPs on the cellular responses than PFPs, Se and PFPs + Se may be related to the better absorption, higher bioavailability, metabolism rates/pathways and intracellular targets. The precise mechanisms underlying Se-PFPs-mediated down regulation of CYP450 enzymes need to be further elucidated.

It is worth noting that administration of mice with Se-PFPs at the levels up to 5.4 g/kg·BW caused no detectable toxic side effects as no significant differences in mean body weight and mean liver index and no death of mice were found among Se-PFPs, PFPs, Se, PFPs + Se and negative control group during the 30-day experiments. However, further measurement of PCE/PCE + NCE is needed to definitely determine whether or not Se-PFPs can cause bone marrow toxicity.

Several previous studies have confirmed that adequate dietary selenium exerts preventive effects on two of the most common types of malignancies prostate and colorectal cancer [61,62,63]. The induction of antioxidant system in liver and anti-mutagenic effects of PFPs + Se observed in this study plus the immuno-modulatory effects observed in our previous study [23,24,41] indicate that PFPs + Se can be an important dietary selenium sources for prostate, colorectal cancer and liver cancer as well.

## 4. Experimental Section

### 4.1. Materials and Chemicals

Cyclophosphamide (CP), mitomycin C (MMC), colchicine, NADPH, resorufin, ethoxyresorufin and methoxyresorufin were purchased from Sigma (St. Louis, MO, USA). Superoxide dismutase (SOD) kit and glutathione peroxidase (GPx) kit were obtained from Jiancheng Bioengineering Institute (Nanjing, China). SYBR Green Master Mix kit was obtained from Qiagen (Venlo, The Netherlands). Nuclear and Cytoplasmic Extraction kit was purchased from BestBio (Shanghai, China). Fetal bovine serum (FBS) was obtained from Gibco (Grand Island, NY, USA). Goat polyclonal anti-mouse CYP1A, HRP-conjugated β-actin were commercially obtained from Santa Cruz Biotech (Dallas, TX, USA). Sodium selenite was purchased from Sigma. Nitrocellulose membranes were obtained from Bio-Rad (Hercules, CA, USA). ECL Plus reagents were obtained from Pierce (Rockford, IL, USA). All the other reagents were of analytical grade.

### 4.2. Preparation of Polysaccharides from Se-Enriched P. fortuneana (Se-PFPs)

*P. fortuneana* fruits were collected from the mountain area in Shiyan, Hubei, China, while Se-enriched *P. fortuneana* fruits were obtained from the mountain area in Enshi, Hubei, China. Se-PFPs were extracted from Se-enriched *P. fortuneana* as described previously [23,24,25]. Briefly, the fruits of *P. fortuneana* were dried in an infrared dryer, and crushed into fine powder by a multifunctional disintegrator. The products were refluxed and degreased twice with 5 L of petroleum ether at 75 °C for 5 h each time. After cooling, the extract obtained was placed into hot water, concentrated under a vacuum at 55 °C and precipitated with 4-fold volume of ethanol at 4 °C overnight. The precipitate was dissolved in Sevag reagent to remove protein, followed by dialysis against distilled water for 48 h. After centrifugation at 2000× *g* for 15 min, the supernatant was pooled, condensed and lyophilized successively to obtain Se-PFPs.

### 4.3. Animals

All of the experimental procedures with animals were conducted in accordance with the standards specified in the 8th edition of Guide for the Care and Use of Laboratory Animals. Kun Ming mice (19–23 g, male:female 50:50) with quality certificated number SYXK 2007-0001 were purchased from Experimental Animal Center of Chongqing Medical University (Chongqing, China) and housed in plastic cages under un-crowded and controlled conditions at the room temperature of 25 ± 2 °C , relative humidity of 50% ± 2% and 12 h light/dark cycle. The mice had free access to water and food. The study was performed after the mice were allowed to acclimate for 1 week.

### 4.4. Experimental Design

**Figure 3:**
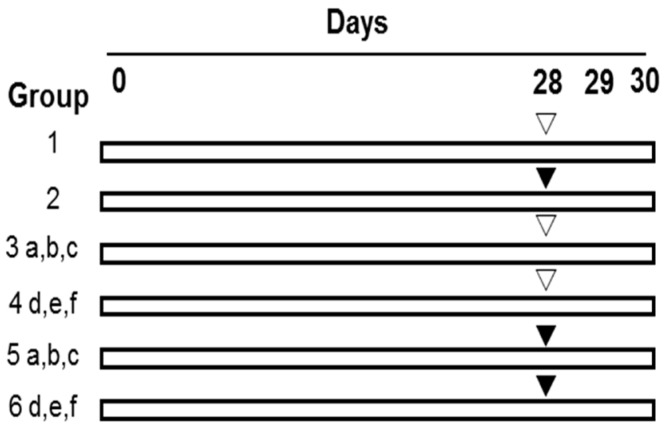
Experimental design to analyze the effect of Se-PFPs on micronuclei formation induced by CP. i.p., intraperitoneally; i.g., by gavage; PFPs, polysaccharide from *P. fortuneana*; Se-PFPs, polysaccharide from Se-enriched *P. fortuneana*; and PFPs + selenite, the combination of PFPs and selenite. ▽: 0.9% NaCl (i.p.); ▼: 40 mg/kg body weight of CP (i.p.); a: 1.35 g of Se-PFPs/kg·BW (i.g.); b: 2.7 g of of Se-PFPs/kg·BW (i.g.); c: 5.4 g of Se-PFPs/kg·BW (i.g.); d: 2.7 g of PFPs/kg·BW (i.g.); e: 10 μg of selenite/kg·BW (i.g.); f: 2.7 g of PFPs/kg·BW + 10 μg of Selenite/kg·BW (i.g.).

As shown in Figure 3, after being weighted, the mice were randomly divided into the 14 groups with 10 mice in each group as follows. In groups 1 (0.9% NaCl) and 2 (CP), the mice in each group were administrated with drinking water for 30 consecutive days and meanwhile intraperitoneally (i.p.) treated with normal saline (0.9% NaCl) and 40 mg of CP/kg body weight (BW), respectively, from Day 28 to Day 30, once a day. In group 3 (Se-PFPs), all of the mice were administrated with Se-PFPs by gavage (i.g.) at respective concentrations, i.e., 1.35 (a), 2.7 (b) and 5.4 (c) g/kg·BW of Se-PFPs for 30 continuous days and simultaneously treated with normal saline once a day by i.p. from Day 28 to Day 30. As the Se content is 3.7 μg/g of Se-PFPs [23,24,25], the Se levels in (a), (b) and (c) were equivalent to 5, 10 and 20 μg/kg·BW, respectively. In group 4, during 30 continuous days, the mice were administrated with 2.7g of PFPs/kg·BW (d), 10 μg of Se/kg·BW (e), or 2.7 g of PFPs/kg·BW + 10 μg of Se/kg·BW (f), once a day by i.g., and meanwhile treated with normal saline once a day by i.p. from Day 28 to Day 30. In group 5, the mice were given the same treatment as that in group 3 for 30 continuous days and simultaneously treated with 40 mg of CP/kg·BW once a day by i.p. from Day 28 to Day 30. In group 6, the mice were given the same treatment as that in group 4 for 30 continuous days and simultaneously treated with 40 mg of CP/kg·BW once a day by i.p. from Day 28 to Day 30.

On Day 30, the mice in each group were weighed at about 6 h after the final treatment. Some of the blood samples were collected by extirpating the eyeballs in heparinized polypropylene tubes and stored at −20 °C. Others were centrifuged for 10 min at 1500× *g*, 4 °C, and stored at −20 °C for the following experiments. After the mice were sacrificed by cervical dislocation, their liver was immediately excised and weighed to evaluate the liver index, which was expressed as the percentage of liver weight (mg) in relative to total body weight (g). Some liver tissue samples were homogenized with dissecting buffer (pH = 7.2, 0.3 mmol/L sucrose, 25 mmol/L imidazole, 1 mmol/L EDTA, 8.5 μmol/L leupeptin, and 1 μmol/L phenylmethylsulfonyl fluoride). After centrifuged at 9000× *g* at 4 °C for 15 min, the supernatant was saved and centrifuged again at 105,000× *g* at 4 °C for 1 h. Then, the precipitate was resuspended and homogenized by dissecting buffer using an ultrasonic homogenizer (UH-50, SMT Co., Ltd., Tokyo, Japan) to obtain the microsomal fraction [64]. The remaining liver tissue was homogenized with 9-fold volumes of cold homogenizing buffer (1 mmol/L EDTA, 0.32 mol/L sucrose and 10 mmol/L Tris-HCl) at pH 7.4. Then, the supernatant was obtained after centrifuged at 14,000× *g* for 30 min at 4 °C and used for the measurement as described previously [24].

### 4.5. Bone Marrow Micronucleus (MN) Assay

Bone marrow MN assay was performed according to the protocol described in literature [65]. Briefly, after the mice were sacrificed, the femur bones of mice were removed and the bone marrow was flushed with 1 mL of FBS and collected into the centrifuge tubes. The mixture was centrifuged at 1000× *g* for 10 min to precipitate the bone marrow cells. After being suspended in FBS, the suspension of the bone marrow cells was dropped and smeared onto glass slides. After being air-dried at room temperature for 24 h, the air-dried slides were fixed in methanol for 10 min, stained with 5% Giemsa for 8 min and observed blindly using a light microscope with an oil immersion objective at ×100-fold magnification. The term, micronucleus, and the means diameter of the small nucleus were no more than a third of the main nucleus in the binuclear cell. The increased MN frequency usually indicates the presence of DNA damage and/or DNA mutation. Approximately 1000 polychromatic erythrocytes (PCEs) were evaluated per slide, where the ratio of micronucleated polychromatic erythrocytes (MNPCEs) to PCEs was scored blindly. A total of 10,000 PCEs were examined to determine the incidence of MNPCEs. The percentage of reduction in MN frequency was calculated according to the following formula [66,67]: Reduction (%) = (frequency of MN in A − frequency of MN in B)/(frequency of MN in A − frequency of MN in C) × 100, where A is the group treated with CP (positive control); B is the group treated with Se-PFPs, PFPs or Se; C is the group treated with normal saline (negative control).

### 4.6. Peripheral Blood MN Assay

Peripheral blood was prepared according to the previously reported protocol [68]. Briefly, blood was collected from tail vein and then placed on an acridine orange-coated glass slide. Erythrocytes were observed using a light microscope. A total of 1000 reticulocytes (RETs) per animal were examined to determine the incidence of micronucleated reticulocytes (MNRETs). The percentage of reduction in MN frequency was also calculated according to the formula described above.

### 4.7. Measurement of Superoxide Dismutase (SOD) and Glutathione Peroxidase (GPx)

The protein level in liver tissue was determined by Bradford method. Using the corresponding commercial kits, the activities of superoxide dismutase (SOD) and glutathione peroxidase (GPx) were measured and expressed as units per mg of protein.

### 4.8. Measurement of Activity of Cytochrome P450 1A (CYP1A)

The activity of 7-ethoxy resorufin deethylase (EROD) representing the activity of cytochrome P450 1A1 (CYP1A1) was determined using ethoxyresorufin as substrate, according to the protocol described previously [69]. In brief, mouse liver microsomes (0.1 mg) were incubated in Tris buffer (0.05 mol/L, pH = 7.4) containing substrate (5 mmol/L ethoxyresorufin) and NADPH (1 mmol/L) at 37 °C for 10 min. The reaction was stopped by adding ice-cold methanol (1 mL), followed by centrifugation at 2000× *g* for 10 min, and then the substrate was detected using Spectramax Gemini XS (Molecular Devices Co., Silicon Valley, CA, USA), whose Excitation and Emission were 530 nm and 590 nm, respectively.

The activity of methoxyresorufin-*O*-deethylase (MROD) representing the activity of cytochrome P450 1A2 (CYP1A2) was determined using methoxyresorufin as substrate, according to the same protocol as the one described above.

### 4.9. RT-PCR Analysis

The isolation of total RNA from mouse liver microsomes and cDNA synthesis were performed using SYBR Green Master Mix kit, according to the protocol described in our previous study [23,24,25]. Briefly, the amplification of cDNA was performed with the following specific primer in triplicate 100 μL reactions per each respective experimental condition: CYP1A1 (forward primer: GACCTTCCGGCATTCATCCT and reverse primer: TCAGACTTGTATCTCTTGTGGTGCT), CYP1A2 (forward primer: GACATGGCCTAACGTGCAG and reverse primer: GGTCAGAAAGCCGTGGTTG) and GAPDH (forward primer: ACCTGACCTGCCGTCTAGAA and reverse primer: TCCACCACCCTGTTGCTGTA). After pre-denaturing at 95 °C for 10 min, the PCR was performed for 40 cycles as follows: denaturing at 95 °C for 15 s, annealing at 60 °C for 60 s and extension at 72 °C for 45 s. GAPDH was applied as the internal control. The PCR amplified product was separated 1% agarose gel electrophoresis at 100 V for 1 h. The gel was stained by ethidium bromide. The intensity of the PCR-amplified CPY1A1 and CYP1A2 as well as GAPDH was quantified by ImageJ software. Intensities of the PCR-amplified CPY1A1 and CYP1A2 bands were normalized to that of GAPDH and expressed in relative to the value of control, which was set as 1.

### 4.10. Western Blot

Total protein extracts from mouse liver microsomes were obtained according to the protocol described in our previous study [23,24,25]. Briefly, total protein extracts from mouse liver microsomes were lysed on ice with lysis buffer (50 mmol/L Tris, 150 mmol/L NaCl, 10 mmol/L ethylenediaminetetra acetic acid, and 1% Triton X-100) and protease inhibitors. The lysates (50 μg/well) were heated with sample loading buffer for 5 min at 95 °C, separated on 12% SDS-PAGE gel and transferred onto the nitrocellulose membranes. Membranes were blocked for 1 h at room temperature with 5% non-fat powdered milk in TBST buffer (10 mmol/L Tris-HCl, 0.15 mol/L NaCl and 0.05% Tween 20) at pH 7.2 and then incubated at 4 °C overnight with the respective primary antibodies of CYP1A or β-actin (dilution 1:200), followed by incubation for 1 h at room temperature with the corresponding secondary antibodies (dilution 1: 5000). β-actin was applied as the internal control. The bands were observed by ECL Plus reagent and their relative intensities compared to those of β-actin were quantified by ImageJ software.

### 4.11. Statistical Analysis

The statistical analyses were performed with SPSS15.0 statistical software package for Windows (SPSS Inc., Chicago, IL, USA). Results were expressed as mean ± SD. The differences between groups were analyzed using Student’s *t*-test and rate comparison using *X*^2^ test. One-way analysis of variance (ANOVA) was used for the comparison of difference among groups. Difference between groups with *p* < 0.05 was considered to be statistically significant.

## 5. Conclusions

Our study showed that Se-PFPs inhibited CP-induced mutagenicity as reflected by its inhibition of MNPECs and MNRETs, and mitomycin C-induced chromosomal aberrations, enhanced the activities of SOD and GPx in mouse liver, reduced the activities of CYP1A1 and CYP1A2 in mouse liver, and reduced the mRNA and protein levels of CYP1A in mouse liver microsome in a dose-dependent manner, suggesting that the anti-mutagenic potential of Se-PFPs is mediated through inhibiting the activity and expression of members of CYP1A family. In addition, the anti-mutagenic potential of Se-PFPs is higher than those of PFPs, Se or PFPs + Se at the same level, indicating the synergistic effects of Se-PFPs on these cellular responses.

## Figures and Tables

**Table 1:** Changes in mean body weight and mean liver index of mice after various treatments for 30 days (*n* = 10).

Group	Body Weight Changes during the Experiment (g)		Increased Body Weight (g)	Liver Index (%)
	Initial Body Weight	Final Body Weight		
0.9% NaCl	25.47 ± 1.86	35.28 ± 2.37	9.81 ± 0.94	4.72 ± 0.23
CP	26.30 ± 1.05	35.47 ± 2.02	9.17 ± 1.45	5.06 ± 0.39
Se-PFPs (a) + NaCl	26.51 ± 1.50	36.27 ± 1.27	10.07 ± 1.04	5.98 ± 0.22
Se-PFPs (b) + NaCl	25.50 ± 1.31	34.93 ± 3.12	9.32 ± 1.84	5.28 ± 0.54
Se-PFPs (c) + NaCl	26.41 ± 1.51	36.43 ± 2.17	10.15 ± 1.21	5.08 ± 0.31
PFPs + NaCl	25.47 ± 1.92	35.10 ± 2.21	9.63 ± 1.62	5.62 ± 0.51
Se + NaCl	25.46 ± 2.06	35.37 ± 1.64	9.91 ± 1.24	5.44 ± 0.47
PFPs + Se + NaCl	26.57 ± 1.35	36.98 ± 2.17	10.42 ± 1.13	5.52 ± 0.65
Se-PFPs (a) + CP	25.93 ± 1.29	35.67 ± 2.14	9.76 ± 1.64	5.37 ± 0.45
Se-PFPs (b) +CP	25.60 ± 2.13	34.92 ± 2.67	9.34 ± 1.88	5.42 ± 0.61
Se-PFPs (c) + CP	25.69 ± 2.40	35.54 ± 1.87	9.88 ± 2.02	4.91 ± 0.69
PFPs + CP	26.60 ± 1.47	36.71 ± 2.24	10.12 ± 1.67	5.25 ± 0.56
Se + CP	26.52 ± 1.42	36.39 ± 2.53	9.87 ± 1.45	5.67 ± 0.46
PFPs + Se + CP	25.89 ± 2.14	35.32 ± 1.77	9.44 ± 2.55	5.38 ± 0.50

CP, cyclophosphamide; PFPs, polysaccharide from *P. fortuneana*; Se, selenite; Se-PFPs, polysaccharide from Se-enriched *P. fortuneana*.

**Table 2:** Frequencies of micronucleated polychromatic erythrocytes (MNPECs) in bone marrow of mice after various treatments.

Group	No. of Animal	Polysaccharide Dose (g/kg·BW)	Se Dose (μg/kg·BW)	No. of Analyzed Cells	MNPEC		Reduction (%)
					No.	%	
0.9% NaCl	10	0	0	10,000	31 ± 3	0.31 ± 0.03	
CP	10	0	0	10,000	624 ± 17	6.24 ± 0.17 ^a^	
Se-PFPs (a) + NaCl	10	1.35	5	10,000	25 ± 5	0.25 ± 0.05	
Se-PFPs (b) + NaCl	10	2.7	10	10,000	26 ± 4	0.26 ± 0.04	
Se-PFPs (c) + NaCl	10	5.4	20	10,000	29 ± 4	0.29 ± 0.04	
PFPs + NaCl	10	2.7	0.108	10,000	28 ± 5	0.28 ± 0.05	
Se + NaCl	10	0	10	10,000	27 ± 4	0.27 ± 0.04	
PFPs + Se + NaCl	10	2.7	10	10,000	25 ± 4	0.25 ± 0.04	
Se-PFPs (a) + CP	10	1.35	5	10,000	281 ± 9	2.81 ± 0.09 ^b^	57.8
Se-PFPs (b) +CP	10	2.7	10	10,000	186 ± 7	1.86 ± 0.07 ^b^	73.9
Se-PFPs (c) + CP	10	5.4	20	10,000	112 ± 10	1.12 ± 0.10 ^b,d^	86.3
PFPs + CP	10	2.7	0.108	10,000	377 ± 10	3.77 ± 0.10 ^b^	41.7
Se + CP	10	0	10	10,000	362 ± 9	3.62 ± 0.09 ^b,c^	44.2
PFPs + Se + CP	10	2.7	10	10,000	253 ± 9	2.53 ± 0.09 ^b,c^	62.6

^a^ *p* < 0.05, compared with 0.9% NaCl; ^b^ *p* < 0.05, compared with CP; ^c^ *p* < 0.05, compared with Se-PFPs (b) + CP; ^d^ *p* < 0.05, compared with Se-PFPs (a) + CP. CP, cyclophosphamide; PFPs, polysaccharides from *P. fortuneana*; Se, selenite; Se-PFPs, polysaccharide from Se-enriched *P. fortuneana*; BW, body weight.

**Table 3:** Frequencies of micronucleated reticulocytes (MNRETs) in peripheral blood of mice after various treatments.

Group	No. of Animal	Polysaccharides Dose (g/kg·BW)	Se Dose (μg/kg·BW)	No. of Analyzed Cells	MNRET		Reduction (%)
					No.	%	
0.9% NaCl	10	0	0	10,000	33 ± 6	0.33 ± 0.06	
CP	10	0	0	10,000	649 ± 19	6.49 ± 0.19 ^a^	
Se-PFPs (a) + NaCl	10	1.35	5	10,000	26 ± 6	0.26 ± 0.06	
Se-PFPs (b) + NaCl	10	2.7	10	10,000	27 ± 5	0.27 ± 0.05	
Se-PFPs (c) + NaCl	10	5.4	20	10,000	31 ± 6	0.31 ± 0.06	
PFPs + NaCl	10	2.7	0.108	10,000	28 ± 5	0.28 ± 0.05	
Se + NaCl	10	0	10	10,000	27 ± 4	0.27 ± 0.04	
PFPs + Se + NaCl	10	2.7	10	10,000	26 ± 5	0.26 ± 0.05	
Se-PFPs (a) + CP	10	1.35	5	10,000	299 ± 10	2.99 ± 0.10 ^b^	56.8
Se-PFPs (b) +CP	10	2.7	10	10,000	198 ± 8	1.98 ± 0.08 ^b^	73.2
Se-PFPs (c) + CP	10	5.4	20	10,000	107 ± 11	1.07 ± 0.11 ^b,d^	87.9
PFPs + CP	10	2.7	0.108	10,000	398 ± 12	3.98 ± 0.12 ^b^	40.7
Se + CP	10	0	10	10,000	381 ± 11	3.81 ± 0.11 ^b,c^	43.5
PFPs + Se + CP	10	2.7	10	10,000	273 ± 10	2.73 ± 0.10 ^b^	61.0

^a^ *p* < 0.05, compared with 0.9% NaCl; ^b^ *p* < 0.05, compared with CP; ^c^ *p* < 0.05, compared with Se-PFPs (b) + CP; ^d^ *p* < 0.05, compared with Se-PFPs (a) + CP. CP, cyclophosphamide; PFPs, polysaccharide from *P. fortuneana*; Se, selenite; Se-PFPs, polysaccharide from Se-enriched *P. fortuneana*; BW, body weight.

**Table 4:** Superoxide dismutase (SOD) and glutathione peroxidase (GPx) activities in the liver of mice after various treatments (*n* = 10).

Group	SOD Activity (U/mg)	GPx Activity (U/mg)
0.9% NaCl	199.3 ± 17.5	241 ± 21.6
CP	83.2 ± 7.9 ^a^	99.7 ± 12.1 ^a^
Se-PFPs (a) + CP	131.4 ± 16.0 ^b^	157.7 ± 19.2 ^b^
Se-PFPs (b) + CP	168.1 ± 5.0 ^b^	198.9 ± 15.2 ^b^
Se-PFPs (c) + CP	185.6 ± 9.2 ^b,^^d^	222.8 ± 10.9 ^b,^^d^
PFPs + CP	132.4 ± 9.3 ^b^	158.9 ± 12.4 ^b^
Se + CP	127.7 ± 12.8 ^b,^^c^	149.2 ± 10.5 ^b,^^c^
PFPs + Se + CP	152.6 ± 12.9 ^b^	183.1 ± 15.5 ^b^

^a^ *p* < 0.05, compared with 0.9% NaCl; ^b^ *p* < 0.05, compared with CP; ^c^ *p* < 0.05, compared with Se-PFPs (b) + CP; ^d^ *p* < 0.05, compared with Se-PFPs (a) + CP. CP, cyclophosphamide; PFPs, polysaccharide from *P. fortuneana*; Se, selenite; Se-PFPs, polysaccharide from Se-enriched *P. fortuneana*.

**Table 5:** Activities of CYP1A and CYP2A in the liver of mice after various treatments (*n* = 10).

Group	EROD Activity (pmol/min/mg Protein)	MROD Activity (pmol/min/mg Protein)
0.9% NaCl	38.17 ± 1.95	61.27 ± 9.29
CP	38.10 ± 3.18 ^a^	61.1 ± 7.93 ^a^
Se-PFPs (a) + CP	28.93 ± 2.36 ^b^	44.00 ± 5.03 ^b^
Se-PFPs (b) + CP	20.97 ± 2.06 ^b,^^d^	26.93 ± 2.68 ^b,d^
Se-PFPs (c) + CP	13.13 ± 3.56 ^b,^^d^	10.00 ± 1.77 ^b,^^d^
PFPs + CP	28.57 ± 1.51 ^b^	43.88 ± 3.45 ^b^
Se + CP	36.97 ± 2.12 ^c^	60.47 ± 5.6 ^c^
PFPs + Se + CP	28.73 ± 2.41 ^b,e^	43.87 ± 3.72 ^b,e^

^a^ *p* > 0.05, compared with 0.9% NaCl; ^b^ *p* < 0.05, compared with CP; ^c^ *p* > 0.05, compared with CP; ^d^ *p* < 0.05, compared with Se-PFPs (a) + CP; ^e^ *p* < 0.05, compared with Se-PFPs (b) + CP. CP, cyclophosphamide; PFPs, polysaccharide from *P. fortuneana*; Se, selenite; Se-PFPs, polysaccharide from Se-enriched *P. fortuneana*.

## Supplementary Materials

Supplementary materials can be accessed at: http://www.mdpi.com/1420-3049/21/12/1731/s1.
