# Supplementary material for: Antimutagenic Effects of Selenium-Enriched Polysaccharides from Pyracantha fortuneana through Suppression of Cytochrome P450 1A Subfamily in the Mouse Liver
Source: Molecules. 2016 Dec 16;21(12):1731. doi: 10.3390/molecules21121731 (PMC6272851; doi:10.3390/molecules21121731)
Supplement: Supplementary file 1 [file molecules-21-01731-s001.pdf]

# Supplementary Materials: Antimutagenic Effects of Selenium-Enriched Polysaccharides from *Pyracantha fortuneana* through Suppression of Cytochrome P450 1A Subfamily in the Mouse Liver

**Fan Peng, Xin Guo, Zhihong Li, Changzheng Li, Changdong Wang, Weiran Lv, Junjie Wang, Fangxiang Xiao, Mohammad Amjad Kamale and Chengfu Yuan**

**Table S1.** Protection against MMC-induced chromosomal aberrations in male mice testicle cells by Se-PFPs, PFPs, Selenite, Selenite + PFPs.

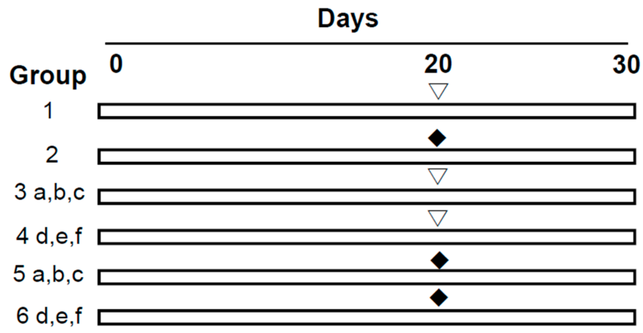


▽: 0.9% NaCl (ip) ◆: 2 mg/kg.bw of MMC (ip)

a: 1.35 g/kg.bw of Se-PFPs (ig)

b: 2.7 g/kg.bw of Se-PFPs (ig)

c: 5.4 g/kg.bw of Se-PFPs (ig)

d: 2.7 g/kg.bw of PFPs (ig)

e: 10 μg/kg.bw of selenite (ig)

f: 2.7 g/kg.bw of PFPs +10 μg/kg.bw of selenite (ig)

| Group | Polysaccharides Dose (g/kg·bw) | Selenium Dose (μg/kg·bw) | No. of Animal | No. of Analyzed Cells | Chromosomal Aberrations | | Inhibition (%) |
|---|---|---|---|---|---|---|---|
| | | | | | No. | % | |
| 0.9% NaCl | 0 | 0 | 10 | 10,000 | 131 ± 13 | 1.31 ± 0.13 | |
| MMC | 0 | 0 | 10 | 10,000 | 1224 ± 83 | 12.24 ± 0.83 [a] | |
| Se-PFPs (a) + NaCl | 1.35 | 5 | 10 | 10,000 | 102 ± 14 | 1.02 ± 0.14 | |
| Se-PFPs (b) + NaCl | 2.7 | 10 | 10 | 10,000 | 99 ± 11 | 0.99 ± 0.11 | |
| Se-PFPs (c) + NaCl | 5.4 | 20 | 10 | 10,000 | 94 ± 12 | 0.94 ± 0.12 | |
| PFPs + NaCl | 2.7 | 0.108 | 10 | 10,000 | 115 ± 18 | 1.15 ± 0.18 | |
| Selenite + NaCl | 0 | 10 | 10 | 10,000 | 124 ± 10 | 1.24 ± 0.10 | |
| PFPs + Selenite + NaCl | 2.7 | 10 | 10 | 10,000 | 116 ± 11 | 1.16 ± 0.11 | |
| Se-PFPs (a) + MMC | 1.35 | 5 | 10 | 10,000 | 632 ± 50 | 6.32 ± 0.50 [b] | 54.2 |
| Se-PFPs (b) + MMC | 2.7 | 10 | 10 | 10,000 | 436 ± 49 | 4.36 ± 0.49 [b,d] | 72.1 |
| Se-PFPs (c) + MMC | 5.4 | 20 | 10 | 10,000 | 213 ± 34 | 2.13 ± 0.34 [b,d] | 92.5 |
| PFPs + MMC | 2.7 | 0.108 | 10 | 10,000 | 817 ± 67 | 8.17 ± 0.67 [b] | 37.2 |
| Selenite + MMC | 0 | 10 | 10 | 10,000 | 752 ± 55 | 7.52 ± 0.55 [b,c] | 43.2 |
| PFPs + Selenite + MMC | 2.7 | 10 | 10 | 10,000 | 534 ± 51 | 5.34 ± 0.51 [b] | 63.1 |

[a] $p < 0.05$, compared with 0.9% NaCl (negative control) group; [b] $p < 0.05$, compared with MMC (positive control) group; [c] $p < 0.05$, compared with Se-PFPs (b) + MMC group; [d] $p < 0.05$, compared with Se-PFPs (a) + MMC group.
